# Bone Mineral Status in Children and Adolescents with Klinefelter Syndrome

**DOI:** 10.1155/2016/3032759

**Published:** 2016-06-16

**Authors:** Stefano Stagi, Mariarosaria Di Tommaso, Cristina Manoni, Perla Scalini, Francesco Chiarelli, Alberto Verrotti, Elisabetta Lapi, Sabrina Giglio, Laura Dosa, Maurizio de Martino

**Affiliations:** ^1^Department of Health Sciences, University of Florence, Anna Meyer Children's University Hospital, 50139 Florence, Italy; ^2^Department of Health Sciences, University of Florence, Careggi Hospital, 50134 Florence, Italy; ^3^Department of Paediatrics, University of Chieti, 66100 Chieti, Italy; ^4^Department of Paediatrics, University of L'Aquila, 67100 L'Aquila, Italy; ^5^Genetics and Molecular Medicine Unit, Anna Meyer Children's University Hospital, 50139 Florence, Italy

## Abstract

*Objective*. Klinefelter syndrome (KS) has long-term consequences on bone health. However, studies regarding bone status and metabolism during childhood and adolescence are very rare.* Patients*. This cross-sectional study involved 40 (mean age: 13.7 ± 3.8 years) KS children and adolescents and 80 age-matched healthy subjects. For both patient and control groups, we evaluated serum levels of ionised and total calcium, phosphate, total testosterone, luteinising hormone, follicle stimulating hormone, parathyroid hormone (PTH), 25-hydroxyvitamin D (25(OH)D), 1,25-dihydroxyvitamin D, osteocalcin, bone alkaline phosphatase, and urinary deoxypyridinoline concentrations. We also calculated the* z*-scores of the phalangeal amplitude-dependent speed of sound (AD-SoS) and the bone transmission time (BTT).* Results*. KS children and adolescents showed significantly reduced AD-SoS (*p* < 0.005) and BTT (*p* < 0.0005)* z*-scores compared to the controls. However, KS patients presented significantly higher PTH (*p* < 0.0001) and significantly lower 25(OH)D (*p* < 0.0001), osteocalcin (*p* < 0.05), and bone alkaline phosphatase levels (*p* < 0.005). Interestingly, these metabolic bone disorders were already present in the prepubertal subjects.* Conclusions*. KS children and adolescents exhibited impaired bone mineral status and metabolism with higher PTH levels and a significant reduction of 25-OH-D and bone formation markers. Interestingly, this impairment was already evident in prepubertal KS patients. Follow-ups should be scheduled with KS patients to investigate and ameliorate bone mineral status and metabolism until the prepubertal ages.

## 1. Introduction

Klinefelter syndrome (KS) is a common chromosomal disorder with a prevalence of 120–153 per 100,000 live-born male births [1, 2]. KS is commonly characterized by the presence of one extra X chromosome in a male phenotype resulting in a 47, XXY karyotype [2, 3]. The major KS finding is represented by primary hypogonadism due to seminiferous tubule dysgenesis and androgen deficiency, resulting in a progressive testicular failure that begins during pubertal development. The clinical phenotype includes increased stature, gynecomastia, small testes, and infertility [4], and the most frequently associated medical disorders can be categorized as follows: (1) motor, cognitive, and behavioural dysfunction; (2) tumours; (3) vascular disease; and (4) endocrine/metabolic and autoimmune diseases [5].

KS is an underdiagnosed condition; in fact, only 25% of patients are diagnosed, with only a minority of those diagnosed before puberty [2, 3, 6], whereas the majority of patients are identified after puberty onset [5, 6].

Testosterone (T) is an important hormone that plays a role especially during puberty to achieve bone maturation and adequate peak bone mass (PBM). In KS metabolic bone disorders have been reported [7, 8] and low testosterone may be responsible for decreased bone mineral density (BMD) [7], since it is involved in bone metabolism regulation directly, through the osteoblast androgen receptor (AR), and indirectly, through aromatization of androgens to oestrogen [8].

In fact, spine BMD in the lumbar region increased significantly after testosterone treatment in KS adults [9]. On the contrary, low bone mass was also observed in patients with normal testosterone levels [10]. Some data seem to demonstrate that the AR CAG polymorphism may contribute to a decreased bone mass [11]. In an interesting study, Aksglaede et al. analysed the muscle/fat ratio and bone mineral content (BMC) in 6 KS boys receiving androgen and 18 KS untreated participants and observed an unfavourable muscle/fat ratio with normal lumbar BMD and whole body BMC in the untreated KS group [12]. Moreover other studies showed that 25-OH-vitamin D levels are reduced in KS adults, highlighting that hypovitaminosis D could be more important than low T levels in inducing low BMD in these patients [7]. Other data demonstrated that the femoral neck BMD improvement after ibandronate therapy was inversely related to 25(OH)D levels [10].

Finally, a recent study showed that KS patients had lower total volumetric BMD, reduced trabecular density, and reduced cortical area and thickness at the tibia, compromising one strength at this site. These data suggest specific bone characteristics related to this genetic syndrome with picture similar to postmenopausal osteoporosis [13].

Studies evaluating the bone status and metabolism of children and adolescents are very rare; however, the limited data available seem to suggest that this unfavourable metabolic bone profile may be present in childhood as well [12].

Therefore, the purpose of our study was to evaluate bone mineral status and metabolism in a cohort of KS children and adolescents.

## 2. Subjects and Methods

Forty nonmosaic KS patients (mean age: 11.96 ± 3.97 years) were recruited consecutively from February 2013 to May 2015 at Anna Meyer Children's University Hospital in Florence, Italy. The cohort contained both children (19 patients, mean age: 8.55 ± 1.39 years) and adolescents (21 patients, 15.03 ± 1.60 years). None of the patients had ever received sex hormone therapy.

The study was conducted according to the Declaration of Helsinki and the European Guidelines on Good Clinical Practice. Ethical approval was obtained from the Ethics Committee of our hospital. Written informed consent was obtained from parents and patients according to age and ability to consent.

### 2.1. Case Definition and Study Protocol

We examined both prenatally diagnosed patients with KS (*n* = 15) and patients diagnosed during childhood and adolescence (*n* = 25). In all patients, chromosomal analysis was performed on peripheral blood lymphocytes, and karyotypes were established on 30 metaphases for each patient. In the presence of a prenatal diagnosis, the karyotype was confirmed postnatally. In some cases, array CGH (44 K array platform Agilent oligonucleotides with a resolution of approximately 100 kb) was also performed. As reported, patients with a mosaic karyotype were excluded.

We recorded clinical and demographic data at the baseline visit, including height, weight, body mass index (BMI), waist circumference (WC), and pubertal staging.

Participants or parents of both the KS patients and controls filled out a questionnaire reviewed by the medical staff during the baseline examination which was related to the pubertal history, the presence or absence of chronic health problems, current and past medications, vitamin D and/or calcium intake, familial and personal bone fracture history and osteoporosis, and physical activity. KS patients and controls showing already-known bone metabolic diseases, hyper/hypoparathyroidism, malabsorptive disorders, chronic renal insufficiency, cancer, and drug addiction (including any medication that could compromise the study such as topic or systemic glucocorticoids, anticonvulsant therapy, calcium, vitamin D supplements in the past six months, sexual steroids, or GnRH analogues) were excluded. None of the participants had a recent history of travelling to warmer, sunnier areas prior to and/or during the study.

Physical activity was assessed with a modified activity score based on the sports/leisure activities (0, <2, or >2 hours per week). Outdoor exposure was quantified through questions regarding each subject's average number of daily outdoor hours and a prospective daily time-activity diary, as previously described [14].

Calcium and vitamin D intake was assessed using the semiquantitative validated Food Frequency Questionnaire [14, 15]. The selection of items was based on the patient's diet, frequency of eating, and relative importance of food items, such as milk and dairy products (including yoghurt, cheese, and chocolate), eggs, meat, fish, cereals, bread, vegetables, and fruits. The subjects were also asked about the portion sizes that were assessed by variously sized models, and each subject chose the model closest to the portion size that he usually consumed, according to Mullen et al. [16].

### 2.2. Vitamin D Status

Serum 25(OH)D levels were stratified according to the brackets, ≤10, 11–20, 21–30, and >30 ng/mL, and were defined as severe deficiency, deficiency, insufficiency, and sufficiency, respectively, according to previously established guidelines for bone health (in the absence of a consensus regarding appropriate levels for endocrine and extraendocrine health) [15, 17].

### 2.3. Quantitative Ultrasound (QUS) Scans

Bone mineral status was evaluated with a DBM Sonic 1200 device (IGEA Bone Profiler, Carpi, Italy). The device was equipped with two probes mounted on an electronic calliper. The emitter probe was positioned on the medial surface, and the receiver probe was positioned on the lateral side of the measured phalanx [17]. The time interval between emission and reception of the ultrasound signal was measured and expressed in m/s. Using this technique, we measured (1) the amplitude-dependent speed of sound (AD-SoS, m/s), which is the interval between the start time of the transmitted signal and the time at which the signal received reached the predetermined minimum amplitude value of 2 mV for the first time, and (2) the bone transmission time (BTT, *μ*s), which is the difference between the transmission time in the phalanx soft tissue and the bone and transmission time in the phalanx soft tissue. AD-SoS and BTT standard deviation scores (SDS) were automatically generated [17].

The final result is the average AD-SoS and BTT over four fingers (digits II–V). All of the QUS measurements were carried out by the same operator on the patients' nondominant hands. The coefficients of precision, in vivo, were 0.8% and 1.5% for AD-SoS and BTT, respectively [17].

Low bone mineral status was defined in the presence of at least one densitometric parameter, such as AS-SoS or BTT ≤ −2.0* z*-scores [17].

### 2.4. Control Group

The data obtained were compared with an age-, sex-, and body-size-matched healthy subject control group (80 subjects, mean age: 12.03 ± 4.12 years). For every patient, we selected two control subjects that matched the following criteria: age ± 6 months, height ± 2.0 cm, weight ± 2.0 kg, and equivalent pubertal stage. Informed consent was obtained from all parents of the subjects. This healthy control group was randomly selected from a population survey of healthy Caucasian inhabitants in Tuscany with no endocrine or metabolic diseases; some of these subjects had been evaluated for noninflammatory musculoskeletal complaints.

### 2.5. Study and Laboratory Methods

Height was measured using a wall-mounted stadiometer, and weight was measured to the nearest 0.1 kg. The measurements were performed by the same trained staff members. We calculated BMI as weight in kilograms divided by height in metres squared (kg/m^2^). Waist circumference (WC) was assessed using inelastic anthropometric tape with a length of 2 m and an accuracy of 1 mm. Measurements were taken at the midpoint between the last rib and the iliac crest. However, height, BMI, and WC were normalised for chronological age by converting to SDS, as previously described [18]. Pubertal staging was carried out according to Tanner and Whitehouse's criteria using an orchidometer [19].

For adolescent patients in the late phase of puberty, hypogonadism was defined as total testosterone (T) < 10.4 nmol/L, and severe hypogonadism was defined as T < 8 nmol/L [20].

All participants were examined in the morning after an overnight fast. Blood samples were collected, and serum and plasma were immediately separated and stored at −20°C in multiple vials for future analysis.

Serum levels of total (normal range (NR) = 2.10–2.50 mmol/L) and ionised calcium (NR = 1.10–1.32 mmol/L), phosphate (NR = 1.4–1.7 mmol/L for children aged 2–12 years and 1.1–1.4 mmol/L for youth aged 12–16 years), creatinine, and albumin were measured by a standard autoanalyser method routinely used for daily practice.

Serum levels of FSH (pubertal NR = 0.7–11.1 IU/L), LH (pubertal NR = 0.8–7.6 IU/L), E2 (pubertal NR = 9.9–24.80 pmol/L), total T (pubertal NR = 2.7–17.3 nmol/L), DHEAS (NR = 80–560 *μ*g/dL), intact PTH (NR = 12–72 pg/mL), and 25(OH)D (NR ≥ 30 ng/mL) were determined by chemiluminescent immunometric assays using commercial available kits for the IMMULITE 2000 Automated Analyser (Diagnostic Products Corporation (DPC), Medical System, Genova, Italy). The intra-assay and interassay coefficients of variation (CVs) in these hormone assays were all less than 8% in the full range.

Serum inhibin B was measured by a specific ELISA (Oxford Bio Innovation, Oxford, UK) with a detection limit of 20 pg/mL. Intra-assay and interassay CVs were less than 13% and less than 19%, respectively.

Serum 1,25(OH)2D was determined according to a competitive binding protein assay (Nichols Diagnostics, San Juan Capistrano, CA, USA). The interassay CV was 8%. The normal range is stated as 19.9–67.0 pg/mL.

A commercially available radioimmunoassay kit was used to measure serum osteocalcin levels (CIS Diagnostici S.p.A., Tronzano Vercellese, Italy). The sensitivity of the method was 0.50 ng/mL.

Urinary deoxypyridinoline concentrations were measured using high-resolution chromatography in a fluid environment (Medical System, Genova, Italy). We expressed deoxypyridinoline values in nM for mM of nocturnal 12 hr urinary creatinine. The intra-assay and interassay CVs were <9.8%.

The serum level of bone-specific alkaline phosphatase (BSAP) was measured by immunoassay (Metra Biosystems, Mountain View, CA, USA) with sensitivity of 0.7 U/L and a CV of 3.9–5.8%.

### 2.6. Statistical Analysis

Statistical analyses were performed using SPSSX (SPSSX Inc., Chicago, IL, USA). Summaries of continuous variables are given as mean ± standard deviation (or median and range, depending on whether the data were normally distributed or not). To compare differences, we used Student's *t*-test and Mann-Whitney *U* test depending on the distribution of the analysed variable. The chi-squared test and Fisher's exact test were used to examine associations between dichotomous variables. Spearman's (rank) correlation test was used to determine the correlation coefficients. We used multiple stepwise regression to determine the variables (age (years), serum PTH concentration, ionised and total calcium, phosphate, BSAP, 25(OH)D and 1,25(OH)2D, osteocalcin, LH, FSH, total testosterone levels, urinary deoxypyridinoline concentration, quantitative assessment of physical activity (hours per week), calcium intake (mg/day), and vitamin D intake (IU/day)) that might correlate independently with AD-SoS* z*-score values. *p* values < 0.05 were considered to be statistically significant.

## 3. Results

Demographic, clinical, and laboratory characteristics of KS patients and healthy controls are summarised in Table 1.

We found no statistically significant differences in height, weight, BMI, WC, and pubertal staging between KS and controls (Table 1). History of fractures was not statistically different between KS and controls (12.5% versus 13.7%). These results were also confirmed considering the prenatally KS diagnosed subgroup and the KS group diagnosed during childhood or adolescence. In addition, no significant differences were found in the KS group for calcium intake (children: 802 ± 267 mg/day; adolescents: 776 ± 211 mg/day) and dietary vitamin D intake (children: 193 ± 67  IU/day; adolescents: 171 ± 49) compared to controls (children: 823 ± 291 mg/day and 179 ± 49  IU/day; adolescents: 779 ± 221 mg/day and 184 ± 54  IU/day; *p* = NS) (Table 1).

**Figure 1:**
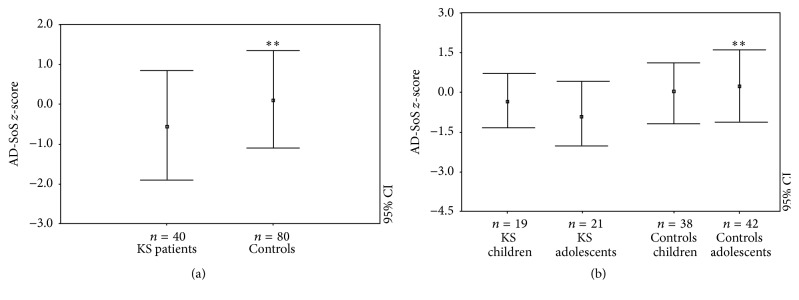
AD-SoS* z*-scores in KS patients and controls (a) and in KS patients and controls when classified by age group (as children and adults) with the relative control groups (b). ^*∗∗*^ *p* < 0.005.

**Figure 2:**
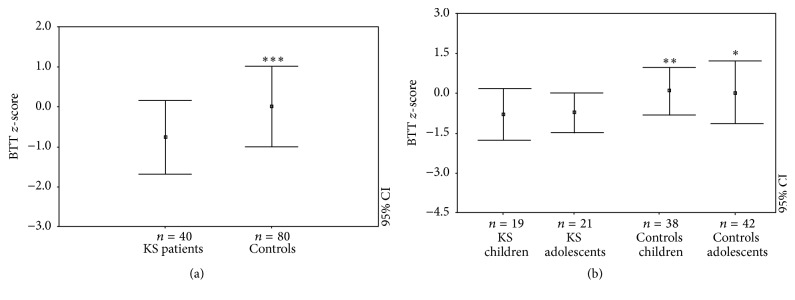
BTT* z*-scores in KS patients and controls (a) and in KS patients and controls when classified by age group (as children and adults) with the relative control groups (b). ^*∗*^ *p* < 0.05; ^*∗∗*^ *p* < 0.005; ^*∗∗∗*^ *p* < 0.0005.

Patients with KS showed significant differences regarding AD-SoS (−0.55 ± 1.13) and BTT* z*-scores (−0.74 ± 0.91) compared to the controls (resp., 0.12 ± 1.23 and 0.02 ± 1.05, *p* < 0.005 and *p* < 0.0005; Figures 1(a) and 2(a)). This same outcome persisted in the BTT* z*-score when KS patients were divided into children (−0.77 ± 0.98 versus 0.13 ± 0.91; *p* < 0.005) and adolescents (−0.71 ± 0.77 versus 0.06 ± 1.23; *p* < 0.05) (Figure 2(b)). On the contrary, the AD-SoS* z*-scores were significantly different between KS adolescents and controls (−0.87 ± 1.27 versus 0.21 ± 1.41; *p* < 0.005) but not between children and controls (−0.28 ± 1.00 versus −0.02 ± 1.05) (Figure 1(b)).

Overall, 7/40 KS patients (17.5%) had a low bone mineral status (*z*-score ≤ −2.0). This incidence was significantly higher than that observed in the control group (2/80: 2.5%; *p* < 0.0001). In particular, AD-SoS had a ≤−2.0* z*-score in 4/40 patients (10% versus 1.25% of controls; *p* < 0.0001) and BTT* z*-scores ≤ −2.0 in 3/40 patients (7.5% versus 1.25%; *p* < 0.0001), respectively. Of these, 6/7 KS patients showing AD-SoS and BTT* z*-score ≤ −2.0 were adolescents, while in KS children group the incidence was significantly lower (28.2% versus 5.3%; *p* < 0.0001).

KS patients did not show statistically different ionised (1.09 ± 0.05 versus 1.11 ± 0.08 mmol/L) and total calcium levels (2.38 ± 0.06 versus 2.35 ± 0.10 mmol/L) and phosphate levels (1.38 ± 0.15 mmol/L versus 1.34 ± 0.20 mmol/L) compared with controls. The ionised and total calcium and phosphate levels did not differ between KS children and adolescents.

**Figure 3:**
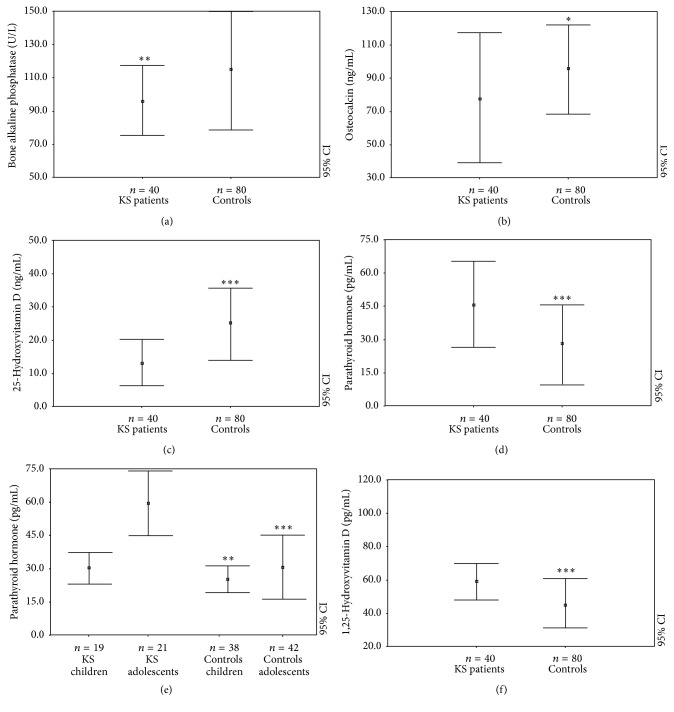
Bone alkaline phosphatase levels (a), osteocalcin serum levels (b), 25-hydroxyvitamin D levels (c), parathyroid hormone levels in total (d) and classified by age (as children and adolescents) (e), and 1,25-hydroxyvitamin D levels (f) in KS patients and controls. ^*∗*^ *p* < 0.05; ^*∗∗*^ *p* < 0.005; ^*∗∗∗*^ *p* < 0.0005.

Furthermore, evaluating bone formation or resorption markers, KS showed reduced levels of BSAP (95.71 ± 17.60 versus 114.50 ± 35.82 U/L, *p* < 0.005) and osteocalcin (78.47 ± 38.72 versus 96.37 ± 26.89 ng/mL, *p* < 0.05) (Figures 3(a) and 3(b)), while we could not find any difference regarding urinary deoxypyridinoline concentrations (Table 1).

The 25-OH-vitamin D levels were significantly lower (13.64 ± 6.48 versus 25.21 ± 11.42 ng/mL, *p* < 0.0001) (Figure 3(c)), whereas PTH levels were significantly higher in KS patients than controls (45.80 ± 18.34 pg/mL versus 26.89 ± 15.37, *p* < 0.0001) (Figure 3(d)). Furthermore, we discovered significant difference regarding PTH levels between children (30.55 ± 6.43 pg/mL) and adolescents (59.59 ± 14.07 pg/mL; *p* < 0.0001) and between KS population and control group (children: 24.89 ± 5.78 pg/mL, *p* < 0.005; adolescents: 29.97 ± 14.56 pg/mL, *p* < 0.0001) (Figure 3(e)). In addition, there was no statistical difference regarding 25(OH)D levels among KS and control subjects. Moreover, evaluating KS patients and controls with the same 25(OH)D levels, the mean PTH values (50.03 ± 17.95 versus 30.25 ± 12.21 pg/mL) and the percentage of hyperparathyroidism (17.5% versus 2.5%, *p* < 0.0001) were significantly higher in KS children and adolescents.

Performing a seasonal analysis (winter = November to April), significantly lower 25(OH)D was found in both periods in KS patients compared to healthy subjects (winter: 8.4 ± 4.25 versus 18.11 ± 7.78, *p* < 0.0001; summer: 16.99 ± 4.88 versus 32.66 ± 8.71, *p* < 0.0001).

Furthermore, we highlighted significant differences regarding 1,25(OH)2D in KS patients (59.35 ± 10.45 pg/mL) and controls (45.90 ± 14.58 pg/mL; *p* < 0.0001) (Figure 3(f)).

At the time of puberty, testosterone serum levels were normal in 10/21 (47.6%) KS adolescents and in the lower normal range in 11/21 (52.4%), whereas LH and FSH were in the normal range in 11/21 (52.4%) KS adolescents and elevated when compared to the normal reference ranges in 10/21 (47.6%) (Table 1). No KS subjects had hypogonadism during the period of the study. Inhibin B was not different if compared to controls in all prepubertal KS patients and in 15/21 (71.4%) KS patients, whereas in 6/21 (28.6%) inhibin B levels were lower than controls. 17*β*-Estradiol levels were significantly different in KS patients and controls (*p* < 0.05), and the ratio between total 17*β*-estradiol and total testosterone (E/T ratio) was significantly higher in KS patients than in healthy controls (data not shown).

A quantitative assessment of physical activity in patients with KS and controls did not reveal significant differences between the two groups: 0 hours per week (34% and 28%, resp.), <2 hours per week (46% and 50%, resp.), and >2 hours per week (20% and 22%, resp.).

Correlation analysis showed that AD-SoS and BTT* z*-scores correlated negatively with PTH (resp., *r* = −0.46, *p* < 0.005; *r* = −0.48, *p* < 0.005) and with age (*r* = −0.62; *p* < 0.0001) and positively with 25(OH)D levels (*r* = 0.47, *p* < 0.005). Both BSAP and osteocalcin levels also showed a significant correlation with total calcium values (resp., *r* = 0.54 and *r* = 0.63; *p* < 0.005). PTH correlated significantly with ionised calcium (*r* = 0.51; *p* < 0.005), age (*r* = 0.61, *p* < 0.0001), total calcium (*r* = 0.48, *p* = 0.002), phosphorus (*r* = 0.35, *p* = 0.04), magnesium (*r* = −0.76, *p* < 0.0001), LH (*r* = 0.66, *p* < 0.0001), FSH (*r* = 0.68, *p* < 0.0001), total testosterone (*r* = −0.66, *p* < 0.0001), vitamin D (*r* = −0.35, *p* = 0.04), 1,25(OH)2D (*r* = 0.37, *p* = 0.02), and osteocalcin (*r* = −0.47, *p* = 0.002).

The multiple regression analysis included age, sex, PTH, ionised and total calcium levels, phosphate levels, 25(OH)D and 1,25(OH)2D levels, serum osteocalcin levels, urinary deoxypyridinoline concentrations, quantitative assessment of physical activity, calcium intake, and BSAP levels and we demonstrated that they are all significant predictors of a lower AD-SoS* z*-score, PTH levels (*p* < 0.001), and 25(OH)D levels (*p* < 0.005).

## 4. Discussion

Our results revealed that KS patients have an impaired bone mineral status that begins early in life. In fact, KS children and adolescents displayed significantly reduced AD-SoS and BTT* z*-scores when compared with healthy subjects. This aspect is particularly concerning since over 17% of KS children and adolescents had* z*-scores ≤ −2.0.

KS children and adolescents also showed an impaired bone metabolism, with significantly reduced 25(OH)D levels and higher PTH levels compared with controls. Finally, KS children and adolescents seem to present reduced levels of bone formation markers, such as BPSA and osteocalcin levels.

The aetiopathogenesis of this impaired bone mineral status in KS patients may be multifactorial, including the specific bone characteristics related to this genetic syndrome and environmental factors, that is, poor vitamin D levels, as reported by previous studies [7, 21]. Particularly, in our study, KS patients had vitamin D and calcium intake levels comparable to those of controls, while their 25(OH)D levels were significantly lower and PTH levels were significantly higher than controls. These observations confirm data on KS adults reported by Ferlin et al. [7] and Bojesen et al. [21], who showed that 25(OH)D levels were significantly lower compared with controls and associated to significantly lower lumbar and femoral BMD. Ferlin et al. also showed higher levels of PTH, while Bojesen, despite important hypovitaminosis D, did not show a significant increase in PTH comparing KS patients to controls. In both studies a reduction of procollagen type 1 N propeptide (P1NP) levels, a marker of bone apposition, was described, confirming our data showing a reduction of osteocalcin and bone alkaline phosphatase levels, which are markers of bone apposition.

In KS adults, testosterone deficiency is reported to be associated with a significant decrease in BMD; this deficiency is considered as the major risk factor for premature osteopenia and osteoporosis [22–25] and is associated with alterated body composition [26, 27].

In KS, this relative androgen deficiency begins after pubertal onset, and since most KS patients show anticipated puberty [28, 29], it could be reasonable to find decreasing BMC. Testosterone is fundamental for bone maturation to reach the PBM at the end of puberty and to keep it during adult life, regulating male bone metabolism both indirectly by aromatization to oestrogen and directly on osteoblasts through the AR, promoting periosteal bone formation [30] and reducing bone reabsorption [31].

It has also been reported that both serum levels of testosterone and LH show a significant association with osteoporosis or fractures [32–34]. Interestingly, LH was directly related to a positive influence on bone metabolism in men: LH receptors are present on osteoblasts, and LH receptor knockout animals showed age-dependent bone loss [35]. This aspect has been hypothesized in untreated adult patients affected by central hypogonadism that, despite similar hormonal levels of adults with primary hypogonadism, displayed significantly lower BMD [36]. Furthermore, recent evidences have highlighted that FSH have profound effects on bone and influence proinflammatory and proosteoclastogenic cytokine expression [37]. In fact, in postmenopausal women, increased FSH serum concentrations induce mRNA expression of genes involved in osteoclastic phenotypes and function, such as* receptor activator of NF-κB* (*Rank*),* tartrate-resistant acid phosphatase* (*Trap*),* matrix metalloproteinase-9* (*Mmp-9*), and* Cathepsin K*, in a dose-dependent manner, probably accelerating bone loss [38].

Therefore, all these factors may have a role in reducing BMD and impairing the bone metabolism in KS patients. Nevertheless, these data do not explain our finding of an impaired bone metabolism also in children with KS, who usually do not have the typical pubertal problems related to their syndrome.

Interestingly, some data showed a possible role of vitamin D pathway in KS. In adults, testosterone acts indirectly on the parathyroid hormone-vitamin D axis, because testosterone deficiency is related to a reduction in renal 1a-hydroxylase activity with a subsequent decrease in 1,25-hydroxyvitamin D concentration, the active form of vitamin D [11]. Moreover, it has been demonstrated that testosterone inhibits PTH dependent osteoclast formation through the androgen receptor but not through the production of intrinsic oestrogen in mouse bone cell cultures [39].

It is well known that vitamin D is also an important factor in bone metabolism in prepubertal subjects and that vitamin D deficiency may play a role in worsening bone mass. Low vitamin D and high parathyroid hormone (PTH) levels are frequent findings in KS patients [7, 21], which have also been confirmed by our results. Furthermore, the same authors have showed that vitamin D supplementation seems to be more effective than T replacement therapy alone in increasing BMD [7]. This study also showed that calcifediol supplementation was characterized by a very significant increase of 25(OH)D levels and a significant reduction of PTH levels, which still remained high. However, this study did not present a control group.

Our data interestingly seems to suggest that PTH levels were significantly higher in KS patients than in controls, even in absence of severe hypovitaminosis D, suggesting that KS patients tend to increase PTH levels more than the general population.

In conclusion, in adults with KS, the hypogonadism, or the reduced testosterone levels, may represent one of the most important causes of reduced bone mineral status. However, possible new causes for this impairment in KS patients may be related to specific mechanisms of KS, such as an important hypovitaminosis D and higher PTH levels, aspects that are also present before the puberty onset. Additional studies are necessary to evaluate the bone metabolism in prepubertal KS patients. However, measures to prevent an impaired bone mineral status, such as ensuring adequate vitamin D intake, must be implemented early in life in KS patients.

## Figures and Tables

**Table 1:** Baseline characteristics of Klinefelter syndrome (KS) patients and controls.

	KS	Controls	*p*
Subjects, number	40	80	—
Age, yrs	11.96 ± 3.57	12.03 ± 4.12	—
Children	8.55 ± 1.39	8.50 ± 1.63	—
Adolescents	15.03 ± 1.60	15.24 ± 1.76	—
Height, SDS	0.92 ± 0.67	0.94 ± 0.73	—
Children	0.86 ± 0.69	0.85 ± 0.66	—
Adolescents	1.06 ± 0.61	1.12 ± 0.79	—
BMI, SDS	0.56 ± 0.86	0.63 ± 0.93	—
Children	0.45 ± 1.00	0.52 ± 0.86	—
Adolescents	0.67 ± 0.71	0.71 ± 1.01	—
WC, SDS	0.52 ± 0.66	0.58 ± 0.83	—
AD-SoS, *z*-score	−0.55 ± 1.13	0.12 ± 1.23	<0.005
Children	−0.28 ± 1.00	0.02 ± 1.05	NS
Adolescents	−0.87 ± 1.27	0.21 ± 1.41	<0.005
BTT, *z*-score	−0.74 ± 0.91	0.02 ± 1.05	<0.0005
Children	−0.77 ± 0.98	0.13 ± 0.91	<0.005
Adolescents	−0.71 ± 0.77	0.06 ± 1.23	<0.05
Calcium intake, mg/day	760 ± 239	805 ± 250	NS
Vitamin D intake	161 ± 43	182 ± 47	NS
Total calcium, mmol/L	2.38 ± 0.06	2.35 ± 0.10	NS
Ionised calcium, mmol/L	1.09 ± 0.05	1.11 ± 0.08	NS
Phosphorus, mmol/L	1.38 ± 0.15	1.34 ± 0.20	NS
PTH, pg/mL	45.80 ± 18.34	27.21 ± 17.82	<0.0001
Children	30.55 ± 6.43	24.89 ± 5.78	<0.005
Adolescents	59.59 ± 14.07	29.97 ± 14.56	<0.0001
BSAP, U/L	95.71 ± 17.60	114.50 ± 35.82	<0.005
Osteocalcin, ng/mL	78.47 ± 38.72	96.37 ± 26.89	<0.05
Urinary deoxypyridinoline, nM/mM creatinine	38.20 ± 23.47	44.78 ± 23.99	NS
25(OH)D, ng/mL	13.64 ± 6.48	25.21 ± 11.42	<0.0001
1,25(OH)2D, pg/mL	59.35 ± 10.45	45.90 ± 14.58	<0.0001
Total testosterone, nmol/L	5.70 ± 5.39	6.24 ± 5.51	—
LH, U/L	4.10 ± 5.34	3.58 ± 4.01	—
FSH, U/L	8.43 ± 18.53	4.05 ± 4.00	<0.05
Inhibin B, pg/mL	90.42 ± 32.02	131.40 ± 25.66	<0.0001
Estradiol, pmol/L	15.34 ± 8.31	11.87 ± 7.27	<0.05
Testicular volume, cc	2.98 ± 1.56	7.82 ± 6.72	<0.0001

BMI: body mass index; WC: waist circumference; 25(OH)D: 25-hydroxyvitamin D; 1,25(OH)2D: 1,25-dihydroxyvitamin D; PTH: parathyroid hormone.
